# Acute inhalation of hypertonic saline does not improve mucociliary clearance in all children with cystic fibrosis

**DOI:** 10.1186/1471-2466-11-45

**Published:** 2011-09-06

**Authors:** Beth L Laube, Gail Sharpless, Kathryn A Carson, Amber Kelly, Peter J Mogayzel

**Affiliations:** 1 Eudowood Division of Pediatric Respiratory Sciences, The Johns Hopkins Medical Institutions, Baltimore, Maryland 21287, USA

## Abstract

**Background:**

Little is known of how mucociliary clearance (MCC) in children with cystic fibrosis (CF) and normal pulmonary function compares with healthy adults, or how an acute inhalation of 7% hypertonic saline (HS) aerosol affects MCC in these same children.

**Methods:**

We compared MCC in 12 children with CF and normal pulmonary function after an acute inhalation of 0.12% saline (placebo), or HS, admixed with the radioisotope ^99 m^technetium sulfur colloid in a double-blind, randomized, cross-over study. Mucociliary clearance on the placebo day in the children was also compared to MCC in 10 healthy, non-CF adults. Mucociliary clearance was quantified over a 90 min period, using gamma scintigraphy, and is reported as MCC at 60 min (MCC60) and 90 min (MCC90).

**Results:**

Median [interquartile range] MCC60 and MCC90 in the children on the placebo visit were 15.4 [12.4-24.5]% and 19.3 [17.3-27.8%]%, respectively, which were similar to the adults with 17.8 [6.4-28.7]% and 29.6 [16.1-43.5]%, respectively. There was no significant improvement in MCC60 (2.2 [-6.2-11.8]%) or MCC90 (2.3 [-1.2-10.5]%) with HS, compared to placebo. In addition, 5/12 and 4/12 of the children showed a decrease in MCC60 and MCC90, respectively, after inhalation of HS. A *post hoc *subgroup analysis of the change in MCC90 after HS showed a significantly greater improvement in MCC in children with lower placebo MCC90 compared to those with higher placebo MCC90 (p = 0.045).

**Conclusions:**

These data suggest that percent MCC varies significantly between children with CF lung disease and normal pulmonary functions, with some children demonstrating MCC values within the normal range and others showing MCC values that are below normal values. In addition, although MCC did not improve in all children after inhalation of HS, improvement did occur in children with relatively low MCC values after placebo. This finding suggests that acute inhalation of hypertonic saline may benefit a subset of children with low MCC values.

**Trial Registration:**

ClinicalTrials.gov: NCT01293084

## Background

Alterations in airway surface liquid (ASL) hydration [1] and mucus with abnormal surface adhesivity and tenacity [2] are thought to lead to impaired mucociliary clearance (MCC) in patients with cystic fibrosis (CF). Although MCC has been shown to be impaired in adult patients with CF [3-5], it is not clear when the impairment begins. In healthy human lungs, inhaled insoluble material, such as bacteria, viruses, antigens and toxins, deposits in the tracheobronchial ASL and is removed from the lung in a matter of hours by MCC. Cough clearance also plays an important role in removing secretions from CF airways and, to some degree, it can compensate for impairment in MCC.

Recently, the therapeutic use of osmotic agents, such as hypertonic saline (HS) has been proposed to aid in mucus clearance in patients with CF [6]. Inhaled HS may increase airway surface fluid, reduce the abnormal surface adhesivity and tenacity of the mucus and improve MCC. In addition, HS may induce cough, thereby promoting cough clearance.

Several studies have shown that an acute inhalation of HS improves MCC in adult patients with CF [3-5]. The use of HS has also been shown to improve lung function and decrease exacerbations in children and adults with CF [7,8]. For this reason, the clinical use of HS has increased dramatically in the past few years [9]. Despite this rapid uptake into clinical practice, there is little information to guide the use of HS in children with CF who have normal pulmonary function. In this study, we hypothesized that children with CF and normal pulmonary function will have MCC values similar to healthy non-CF individuals and will show significant improvement in MCC following an acute inhalation of HS, compared to placebo.

## Methods

### Study Design and Subject Populations

This was a double-blind, randomized, placebo-controlled study, consisting of a screening visit and two study visits. The protocol was approved by the Johns Hopkins University Institutional Review Board (IRB). Written informed consent was obtained from parents and either written, or verbal assent, was obtained from the participants depending on their age.

Inclusionary criteria were: males and non-pregnant females, age 7-14 years, diagnosis of CF by sweat chloride > 60 meq/L, or presence of two disease-causing *CFTR *mutations, and FEV_1 _≥ 90% of predicted values.

A group of healthy non-CF adults, who had undergone MCC measurements previously at Johns Hopkins, as part of a multicenter MCC standardization protocol, served as a comparison control group. Clearly, healthy children without CF would have been the preferred control group for these studies. However, our IRB does not allow healthy children to be exposed to ionizing radiation for research purposes.

### Concomitant Medications

Children who were taking inhaled tobramycin were studied during the off-month of treatment. Children were asked to stop bronchodilators, rhDNase (dornase-alpha), and airway clearance therapy for 12 hours before and during each study visit.

### Sputum Induction

On the screening visit, children underwent a standardized sputum induction procedure, utilized by the CF Therapeutic Development Network [10]. Children inhaled 3% saline for 12 min using a DeVilbiss Ultra-Neb 99 nebulizer (Sunrise Medical, Somerset, PA). Bacterial and fungal cultures were carried out with any sputum obtained. If sputum was not expectorated, an oropharyngeal culture was obtained. None of the children spontaneously produced sputum at baseline.

### Placebo and Hypertonic Saline Administrations

At least one week following the screening visit, children inhaled 5 ml of 0.12% saline (placebo) on one study visit. During a second study visit, they inhaled 5 ml of 7% HS. The order of these two visits was randomized and they occurred at least one week apart. Placebo and HS aerosols were delivered by an LC Star^® ^nebulizer and ProNeb Ultra^® ^compressor (PARI Respiratory Equipment, Richmond, VA). Prior to the administration of HS or placebo, children inhaled 2 puffs of albuterol (Ventolin^® ^CFC, GlaxoSmithKline, London, England) delivered using an Aerochamber^® ^spacer (Trudell Medical International, London, Ontario). Albuterol was administered to minimize the development of bronchospasm and coughing that has been reported following the administration of HS to patients with CF [11].

We made no attempt to blind the taste of the trial solutions. This was based on advice from our Research Pharmacy, whose staff felt that the addition of any masking agents to the solutions to be aerosolized could have unknown effects on MCC.

### Mucociliary Clearance Measurements

Following inhalation of HS or placebo, patients inhaled the radioisotope ^99 m^technetium sulfur-colloid (radioaerosol) and underwent sequential imaging with a gamma camera (ZLC, Siemens, Gammasonics, Des Plains, IL). Radioaerosol was delivered by an LC Plus^® ^nebulizer (PARI) and DeVilbiss Pulmo Aide^® ^compressor (Sunrise Medical). Children inhaled 20-25 times from the nebulizer starting near functional residual capacity at a flow rate of 0.5 L/sec. During inhalation, the nebulizer was pulsed for a fixed time of 0.7 sec. After inhalation to total lung capacity, children exhaled immediately. There was no breath hold.

Lung images were acquired every 2 min for the first 20 min and every 10 min for up to 90 min. Between the 60 and 90 min measurements, children coughed 30 times in two 15 cough sequences. Clearance values reported at 60 min represented removal of radioisotope and mucus from the airways due to MCC, while values at 90 min represented a combination of MCC and cough clearance. We ensured that all study patients coughed with similar effort by monitoring their peak expiratory flow during each cough with a PiKo-1 electronic peak flow meter (Ferraris Medical, Inc., Louisville, CO).

Lung images were analyzed in terms of the amount of radioactivity initially deposited in the right lung at time 0 (i.e. first lung image) and the amount of radioisotope that was retained at each time point thereafter, using a Sopha computer (SMV, Twinsburg, OH). Counts in the right lung were corrected for time decay and background activity and expressed as a percentage of the radioactivity detected at time 0. Percent clearance was calculated as the difference between percent radioactivity in the lung at time 0 and radioactivity remaining at any given time point thereafter.

The central to peripheral (C:P) ratio was determined from the time 0 image using methods that have been described previously [12]. Larger C:P ratios indicated greater deposition of the radioaerosol in the larger, central airways.

### Sample Size Estimate

Sample size was estimated using Stata Version 9 (StataCorp, College Station, TX) and was based on MCC data from prior studies in adolescent and young adult patients with CF who were treated with HS [5] and adult healthy subjects who were treated with placebo [13]. In the former study [5], the mean difference in MCC at 1 hour was 4.7% (post HS - baseline values). In the latter study [13], mean MCC ± standard deviation (SD) at 76 minutes was 12.3 ± 8.3 [13]. Since our proposed analysis was a paired comparison of differences within the same child, we assumed a range of values for the SD of the difference in clearance between placebo and HS that was less than the variability of independent measurements observed in these two studies. We determined that a sample size of 12 children would provide 80% power to detect a 5% or larger mean difference in MCC between inhalation of placebo versus HS aerosol, assuming the SD of the paired differences was 6% and assuming a = 0.05 (2-sided). A 5% mean difference between treatments is similar to what was reported by Donaldson et al. [5].

### Statistical Analysis

Because of the small subject population, non-parametric analyses were used throughout. Data are presented as box plots showing the median and interquartile range (IQR). The "whiskers" represent the 10^th ^and 90^th ^percentiles. Clearance values are expressed as percent MCC at 60 min (MCC60) and at 90 min (MCC90) after inhalation of the radioaerosol. MCC and C:P ratios for the children on the placebo visit and for the adults were compared using a Wilcoxon rank sum test. The change in MCC60 and MCC90 with HS from placebo in the children was compared using a Wilcoxon signed rank test. In summarizing the results, it was noticed that some children had lower percent MCC values on the placebo visit than others. A *post hoc *subgroup analysis on the change in MCC60 and MCC90 with HS from placebo was conducted using a median split of MCC60 and MCC90 on placebo to compare children with lower MCC to higher MCC using a Wilcoxon rank sum test. C:P ratios in these subgroups were also compared using a Wilcoxon rank sum test. Non-parametric tests were conducted using SAS version 9.22 (SAS Institute, Inc., Cary, North Carolina). All tests were 2-sided and significance was set at P < 0.05.

## Results

### Participant Characteristics

Twelve children completed the screening visit and the two study visits. Their characteristics are shown in Table 1. Three additional children were enrolled, but failed the screening visit. Two other children dropped out after the first study visit. All screening failures and dropouts were replaced. Airway cultures from 10 (83.3%) children grew at least one organism and cultures from 5 children (41.7%) grew 2 organisms.

**Table 1:** Characteristics of Children that Completed the Study

Characteristic	No. (%) or Median (IQR)
No. of patients	12 (100)
Age (years)	10.5 (8.9 - 12.4)
FVC (% predicted)	107 (101.5 - 116.0)
FEV_1 _(% predicted)	108 (102.5 - 117.5)
Height (percentile)	36.5 (16.0 - 70.5)
Weight (percentile)	39.5 (27.5 - 71.5)
BMI (percentile)	48.5 (35.0 - 69.5)
Genotype:	
F508del homozygous	9 (75%)
F508del heterozygous	2 (17%)
Other	1 (8%)
Routine Medications:	
Albuterol	3 (25%)
rhDNase (dornase alpha)	11 (92%)
Salmeterol/fluticasone	7 (58%)
Azithromycin	5 (42%)
Inhaled tobramycin	2 (17%)
Organisms cultured at screening:	
*Staphylococcus aureus*	5 (42%)
*Aspergillus fumigates*	3 (25%)
*Normal/Mixed Respiratory Flora*	2 (17%)
*Pseudomonas aeruginosa*	2 (17%)
*Haemophilus influenza*	2 (17%)
*MRSA*	1 (8%)
*Stenotrophomonas multiphilia*	1 (8%)
*Paecilomyces variotll*	1 (8%)

Abbreviation: IQR, interquartile range

The control group consisted of 10 non-CF adults (6 male, 4 female) without respiratory disease. Their ages ranged between 18-37 years. Median (IQR) age was 24 (22-27) years. Median FEV_1 _was 103% (88-110) predicted. Median FVC was 93% (89-95) predicted.

### Deposition Pattern on Placebo Visit

Median C:P ratio on the placebo visit for all the children was 1.67 (1.29-2.11), which was similar to the healthy adults with a median of 1.71 (1.28-2.06).

### Mucociliary Clearance on Placebo Visit

Median MCC in the children with CF on the placebo visit was 15.4% (12.4-24.5%) at 60 min and 19.3% (17.3-27.8%) at 90 min. These values were not significantly different from those obtained from the 10 healthy, non-CF adults, whose median MCC was 17.8% (6.4-28.7%) and 29.6% (16.1-43.5%) at 60 and 90 min, respectively. Nevertheless, 10/12 children showed MCC90 values less than the median observed in the adults.

### Effect of Hypertonic Saline on Mucociliary Clearance

**Figure 1:**
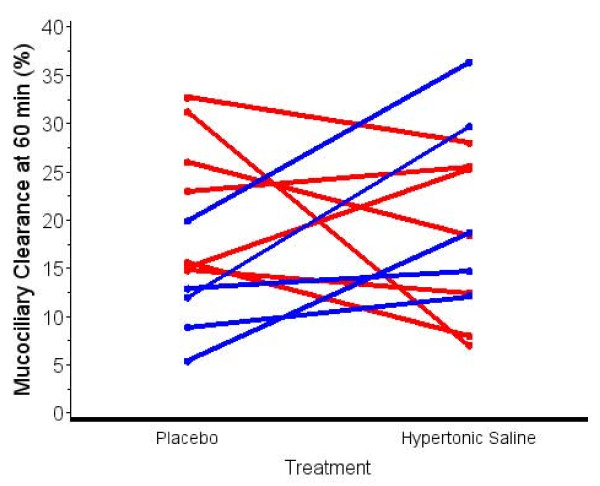
**Effect of hypertonic saline inhalation on mucociliary clearance in individual children (Red - Female patients; Blue - Male patients)**.

**Figure 2:**
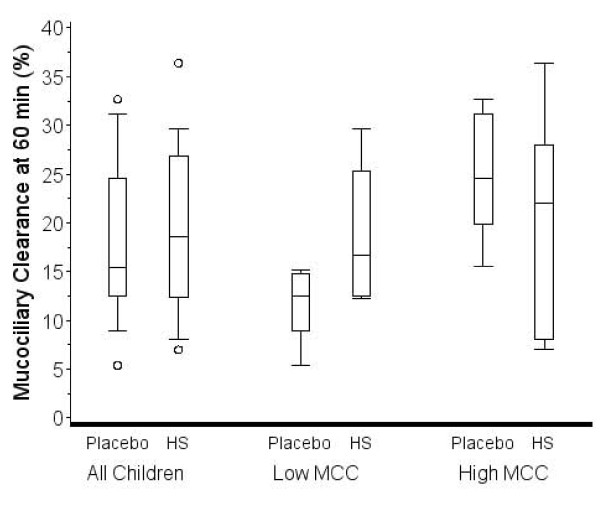
**Box plots showing median and interquartile range for mucociliary clearance at 60 min on the placebo and HS visits for all children and for subgroups of children with less than or greater than the median MCC60 after placebo**. The "whiskers" represent the 10^th ^and 90^th ^percentiles. Open circles represent data outside the 10^th ^and 90th percentiles. The change in MCC with HS was not significantly different from placebo for all children, or for the subgroup comparison.

**Figure 3:**
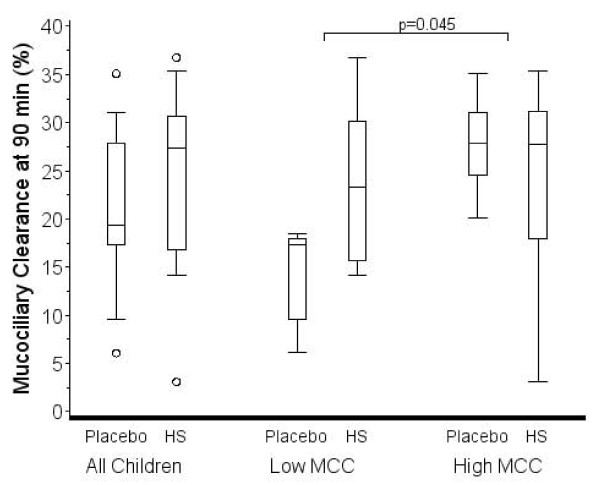
**Box plots showing median and interquartile range for mucociliary clearance at 90 min on the placebo and HS visits for all children and for children with less than or greater than the median MCC90 after placebo**. The "whiskers" represent the 10^th ^and 90^th ^percentiles. Open circles represent data outside the 10^th ^and 90th percentiles. The change in MCC with HS was not significantly different from placebo for all children, but was significantly different for the comparison of the subgroups (p = 0.045).

Figure 1 shows MCC60 after placebo and HS inhalation for each child. Five of twelve children showed a decrease in MCC60 after inhalation of HS. Four of twelve children demonstrated a decrease in MCC90 after inhalation of HS (data not shown). Figure 2 shows the distribution of MCC60 for all children post placebo and HS. The median change on HS compared to placebo was 2.2% (-6.2-11.8%; p = 0.62). Figure 3 shows the distribution of MCC90 for all children post placebo and HS. The median change was 2.3% (-1.2-10.5; p = 0.32).

### *Post Hoc *Analysis: Change in Mucociliary Clearance with HS Compared to Placebo in Children with Low Versus Higher MCC

The distribution of MCC60 and MCC90 for the subgroups after placebo and HS are presented in Figures 2 and 3, respectively. The median change in MCC60 for the lower MCC60 group was 6.8 (1.8-13.3) and in the higher group was -6.2 (-7.6-2.6). The difference in the groups did not reach statistical significance (p = 0.09). The median change in MCC90 for the lower MCC90 group was 10.0 (4.6-13.3) and in the higher group was -1.2 (-3.9-0.0). The difference in the groups was statistically significant (p = 0.045).

### *Post Hoc *Analysis: Aerosol Deposition Pattern in Children with Low Versus Higher MCC on Placebo

The initial aerosol deposition pattern for children with lower MCC60 on the placebo visit was similar to that of children with higher MCC60 on the placebo visit. Median C:P ratio was 1.71 (1.21-2.27) and 1.67 (1.36-1.94) for children with lower and higher MCC60 on the placebo visit, respectively (p = 0.94).

## Discussion

We undertook the current study to quantify MCC in children with CF who have normal pulmonary function and to determine if HS improves MCC. We found that median percent MCC60 (15.4%) and MCC90 (19.3%) on the placebo visit in all children with CF and normal lung function was similar to that of the healthy, non-CF adults. Nevertheless, 10/12 children showed MCC90 values less than the median of 29.6% observed in the adults. These data suggest that percent MCC varies significantly between children with CF lung disease and normal pulmonary functions, with some children demonstrating MCC values within the normal range and others showing MCC values that are below normal values.

These findings are consistent with previous results reported by Mortensen et al. in separate studies of children with CF and healthy adults [14,15]. Mean whole lung clearance at 60 min was 21.1 ± 13.3% in 10 children with CF (11 ± 2 yrs) who were treated with a metered dose inhaler containing the placebo for terbutaline (i.e. no drug) [14]. Median peripheral lung zone clearance at 120 min was 18% (range 12-28%) in 10 healthy subjects who were treated with the same placebo (median age = 30.5 yrs; range = 22-63 yrs) [15]. Nasal MCC also appears to be similar in children with CF and children with no respiratory disease. McShane et al. [16] demonstrated that median nasal MCC was 11 min (range 8-15) in 18 children with CF (median age = 11 yrs; range = 9-15 yrs), compared to 11 min (range 9-16) in 21 children (median age = 12 yrs; range = 9.5-16) with no respiratory disease.

Compared to placebo, an acute inhalation of hypertonic saline did not significantly improve MCC in children in this study. This finding is in contrast to previous studies in which adults with CF demonstrated an improvement in MCC after an acute inhalation of HS [3-5]. However, patients in those studies had significantly worse lung function than the majority of children in this study and such patients may respond differently to HS.

The notion that some patients with CF may respond differently to HS than others is also suggested by the significant improvement in MCC90 after HS in children with lower MCC90 on the placebo visit in this study, compared to children with higher MCC90 values after placebo (Figure 3). It is also interesting that all 5 males in the study showed improvement in MCC60 after HS (Figure 1: blue data lines) and in MCC90 (data not shown). In addition, all 5 males had MCC90 values less than the median split value of 19.3% on the placebo visit. In contrast, only 2 of 7 female children showed improvement in MCC60 (Figure 1) and MCC90 (data not shown) after HS, while 6 of 7 female children had MCC90 values greater than the median split on the placebo visit. Additional studies are needed to explore the effect of HS on patients with low MCC after placebo and the observed gender differences in MCC and possible effects of HS.

Another possible explanation for the observed differences in MCC90 after HS, compared to placebo, for children with lower versus higher MCC90 could involve where the radiolabeled aerosol initially deposited in the lungs. Aerosol that initially deposits in the larger, central airways will be cleared faster than aerosol that is deposited more peripherally because the transit path for removal from the lungs is shorter. However, the initial C:P ratio, an indicator of deposition pattern within the lung, was similar for children with low and higher MCC values. Therefore, differences in the site of deposition cannot account for the observed differences in MCC.

One explanation for the lack of improvement in MCC in more children in our study may be that a single dose of HS is inadequate to show an MCC benefit. In the study of Donaldson et al. [5], MCC improved in patients who were treated 4 times a day with HS for 2 weeks. Similarly, Elkins et al. [7] reported that FEV_1 _rose over four weeks of therapy with HS, implying a cumulative benefit of therapy. Similar results have been seen in early trials of Denufosol, a therapy designed to increase airway surface liquid hydration [17].

Also other measurements may be better at detecting the effects of HS in children with CF who have normal pulmonary function. This hypothesis is suggested by the results of a recent study by Amin and colleagues [18], who used lung clearance index (LCI), a measure of ventilation non-homogeneity. They studied 19 CF patients with a mean age of 10.5 ± 3.1 years and a mean FEV_1 _of 96 ± 12% predicted and found a significant improvement in LCI after 4 weeks of HS compared to treatment with isotonic saline. Again, this significant improvement could have been the result of a longer treatment time, compared to our acute treatment study. It could also be that LCI is a more sensitive indicator of the treatment effect of HS in children with mild CF lung disease than MCC.

There are a few limitations to our study. First, our sample size calculation, based on a paired t-test with a known standard deviation of 6, resulted in a sample size of 12 subjects to have 80% power using a two-sided test with Type I error set at 0.05. However, the observed standard deviation was 12 and for this reason the study was likely underpowered. Secondly, it is possible that some of the patients may have become unblinded, since the taste of HS was salty compared to the placebo. However, the MCC measurements should not have been affected by this differentiation, since MCC measurements are involuntary and do not depend on the patient's effort. Also, the person who performed the data analysis was blinded to the administered solutions. Another limitation is that 10/12 children grew at least one organism after culturing their lung sputum and those organisms may have affected MCC. However, studies of MCC in children infected with specific organisms have not been performed, so the possible affect of these organisms on MCC in children in this study cannot be evaluated. Another limitation was that our control group was an adult population, rather than healthy children. However, their MCC was similar to our CF sample and was consistent with other studies. Finally, we only administered one dose of HS, which may not have been sufficient to elicit significant improvement in MCC in some patients.

## Conclusions

These data suggest that percent MCC varies significantly between children with CF lung disease and normal pulmonary functions, with some children demonstrating MCC values within the normal range and others showing MCC values that are below normal values. In addition, although MCC did not improve in all children after inhalation of HS, improvement did occur in children with relatively low MCC values after placebo. This finding suggests that acute inhalation of hypertonic saline may benefit a subset of children with low MCC values.

## List of Abbreviations

ASL: Airway surface liquid; CF: Cystic fibrosis; FEV_1: _Forced expiratory volume in one second; FVC: Forced vital capacity; HS: Hypertonic saline; MCC: Mucociliary clearance; MCC60: MCC at 60 min; MCC90: MCC at 90 min; CP: Central to peripheral ratio; LCI: Lung clearance index.

## Competing interests

The authors declare that they have no competing interests.

## Authors' contributions

BL and PM conceived of the study, planned its design and coordination and drafted the manuscript. GS performed the mucociliary clearance studies, including image acquisition and processing and data analysis. KC performed the statistical analysis and helped to draft the manuscript. AK performed the induced sputum testing. All authors read and approved the final manuscript.

## Pre-publication history

The pre-publication history for this paper can be accessed here:

http://www.biomedcentral.com/1471-2466/11/45/prepub
